# Effect on healthcare utilization and costs of spinal manual therapy for acute low back pain in routine care: A propensity score matched cohort study

**DOI:** 10.1371/journal.pone.0177255

**Published:** 2017-05-15

**Authors:** Jochen Walker, Ulf Kai Mertens, Carsten Oliver Schmidt, Jean-François Chenot

**Affiliations:** 1 Elsevier Health Analytics, Berlin, Germany; 2 Department of Quantitative Research Methods, Institute of Psychology, University of Heidelberg, Heidelberg, Germany; 3 SHIP-KEF, Institute for Community Medicine, University Medicine Greifswald, Greifswald Germany; 4 Department of General Practice, Institute for Community Medicine, University Medicine Greifswald, Greifswald, Germany; Mayo Clinic Minnesota, UNITED STATES

## Abstract

Spinal manual therapy (SMT) is a popular treatment option for low back pain (LBP). The aim of our analysis was to evaluate the effects of manual therapy delivered by general practitioners and ambulatory orthopedic surgeons in routine care on follow up consultations, sick leave, health service utilization and costs for acute LBP compared to matched patients not receiving manual therapy. This is a propensity score matched cohort study based on health claims data. We identified a total of 113.652 adult patients with acute LBP and no coded red flags of whom 21.021 (18%) received SMT by physicians. In the final analysis 17.965 patients in each group could be matched. Balance on patients’ coded characteristics, comorbidity and prior health service utilization was achieved. The provision of SMT for acute LBP had no relevant impact on follow up visits and days of sick leave for LBP in the index billing period and the following year. SMT was associated with a higher proportion of imaging studies for LBP (30.6% vs. 23%, SMD: 0.164 [95% CI 0.143–0.185]). SMT did not lead to meaningful savings by replacing other health services for LBP. SMT for acute non-specific LBP in routine care was not clinically meaningful effective to reduce sick leave and reconsultation rates compared to no SMT and did not lead to meaningful savings by replacing other health services from the perspective of health insurance. This does not imply that SMT is ineffective but might reflect a problem with selection of suitable patients and the quality and quantity of SMT in routine care. National Manual Medicine societies should state clearly that imaging is not routinely needed prior to SMT in patients with low suspicion of presence of red flags and monitor the quality of provided services.

## Introduction

Spinal manual therapy (SMT) is a popular treatment option for acute low back pain (LBP). A Cochrane Review assessing 20 randomized controlled trials concluded that spinal manual therapy is not more effective for acute LBP compared to inert interventions, sham interventions or as adjunct treatment. The trials were of moderate and low evidence [1]. Consequently most clinical guidelines for management of LBP do not recommend SMT [2, 3, 4], but some consider SMT as an optional treatment [5, 6]. SMT is not a uniform well defined intervention, it comprises various techniques of mobilization (without thrust) and manipulation (with thrust) as well as soft tissue techniques which are often combined based on clinical findings guiding the selection of one or more SMT techniques. A recent more comprehensive review differentiating between acute and chronic LBP and manipulation and mobilization found strong evidence of effectiveness for manipulation alone in patients with acute LBP compared to sham SMT or care as usual and moderate evidence for combination of manipulation and mobilization with care as usual [7]. This review included 5 trials of which only one [8] was included in the previous Cochrane Review. Both reviews observed a high heterogeneity of included patients and of observed outcomes, which might explain the conflicting conclusions. A randomized controlled trial not included in the reviews observed a statically significant reduction in disability with 4 sessions of SMT compared to care as usual which did not reach the minimum clinically important difference [9].

In Germany, SMT provided by physicians certified in manual therapy is covered by the statutory health insurance. Additional Postgraduate training in manual therapy consists of 320 hours on top of completed medical school (6 years of training) and completed specialty training (3–6 years of training). A theoretical and practical examination is required for certification [10]. For billing privileges an additional examination from the regional medical board is required. Virtually all ambulatory orthopedic surgeons and roughly 8% of all general practitioners (GPs) are certified to perform and bill SMT. SMT can be billed twice within a three month billing period. This is fundamentally different from most trials on SMT where the intervention is delivered by chiropractors of physiotherapist with different training and scope of practice [1, 7].

Additionally, patients can be referred to physiotherapy for manual therapy. By law, physiotherapists in Germany are limited to perform mobilization, but are not allowed to perform manipulations with thrust. Although non-physician chiropractors or osteopaths exist in Germany, they only play a minor role in patient care compared to other countries and are mostly not covered by statutory health insurance unlike in other countries. They are not allowed to refer for or perform medical imaging procedures.

Traditionally, imaging was recommended by Manual Medicine Societies to rule out metastasis or fracture prior to manipulations [11]. Given the lack of evidence regarding the clinical utility of routine imaging for acute LBP this is not recommended by clinical guidelines [4, 5, 6]. We hypothesized that SMT for acute LBP might still be associated with increased use of imaging services. Guidelines do not recommend physiotherapy for acute LBP within the first four to six weeks [2, 3, 4, 5, 6], however early referrals are common in practice. We hypothesized that effective SMT provided by trained physicians would decrease the need for pain medication, reduce sick leave, and referrals for physiotherapy. We also hypothesized that using SMT as one complementary alternative treatment option in ambulatory care might decrease the additional use of acupuncture. Therefore manual therapy might increase as well as decrease health service utilization and direct health care costs. A systematic review of cost effectiveness studies, mostly from the United Kingdom concluded that provision of SMT is cost-effective from the health sector perspective [12].

The aim of our analysis was to evaluate the effects of SMT in patients with acute LBP delivered by physicians on follow up consultations, sick leave, health service utilization and total health care costs compared to matched patients not receiving manual therapy in routine care. As analyses are based on health claims data our focus is mainly from the health sector perspective.

## Material and methods

### Design and intervention

**Fig 1:**
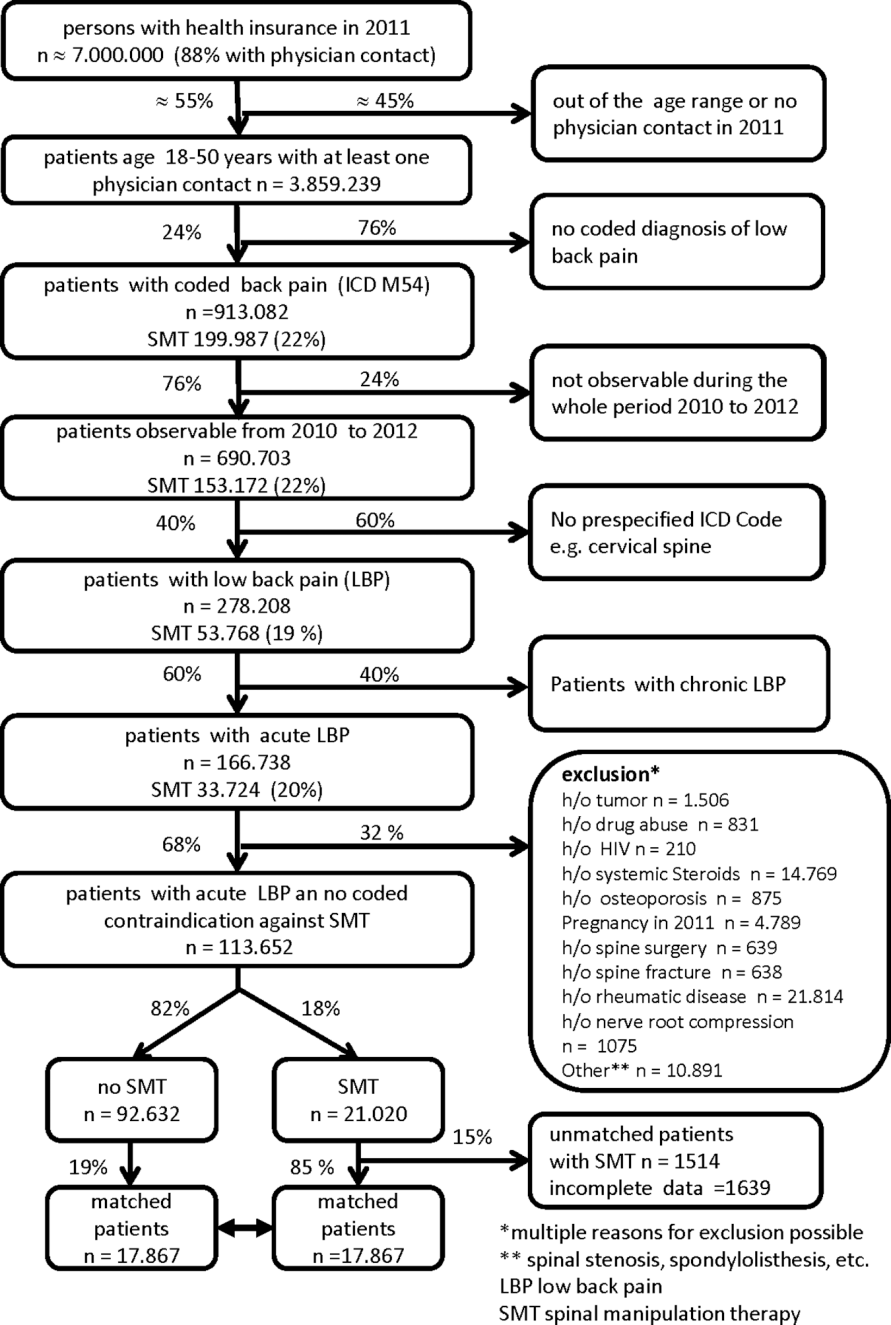
Study flow chart—selection of patients, cases and controls.

We performed a propensity score matched cohort study. The cohort was identified from a health claims data database hosted by the Health Risk Institute (HRI). The database consists of anonymized health claims data from 84 statutory health insurance companies covering seven million insured persons in Germany [13]. For the study a subset of the data was used as described in **Fig 1**. The available data for analysis consists of anonymized basic demographic data, outpatient diagnoses (ICD-10 codes), outpatient billing codes (EBM-GOP), days of sick leave, medication data (ATC-codes), prescription data for physiotherapy and hospital admission data. Patients receiving SMT by licensed physicians were identified with the billing code GOP 30201 [14]. There is only as single umbrella billing code for SMT which does not allow making any detailed inference on the technique used by the billing physician.

We only considered patients who were insured at least for four three-month billing periods (one year) prior to matching and for four three-month billing periods after the index billing period with consultation in the index billing period for LBP for further analysis.

### Patient selection and eligibility criteria

We identified patients consulting with acute LBP in 2011, aged 18 to 50 years. We restricted analyses to patients with new episode LBP episodes, since shorter duration of pain was associated with increased likelihood of successful spinal manipulation [15, 16, 17, 18]. A new episode of LBP was defined as two three-month billing periods without consultation for LBP prior to the index consultation [19, 20]. It is possible that patients with chronic LBP consulting for the first time are misclassified as suffering from acute LBP. Non-specific LBP was defined as one of the following ICD-Codes: M54.15–19, M54.5, M54.85 to 89, M54.95–99. The age restriction was chosen since sick leave was one of our primary outcome measures and we wanted to reduce the probability of other major factors interfering with pain and pain recovery. Age above 50 has been suggested as a red flag and older people are generally considered less suitable for SMT [15,16, 17, 18].

To avoid contamination with patients who either should not receive SMT or suffer from chronic conditions we excluded patients with coded warning signs “red flags” for potentially serious causes of LBP [4, 5, 6]. This includes documented radiculopathy, intravenous drug abuse or opiate replacement therapy, history of cancer, osteoporosis, immunosuppressive therapy, rheumatic diseases including spondylathropathies, vertebral fractures, spine surgery, chronic pain, somatoform pain disorder, treatment in pain clinics, in-patient hospitalization for LBP and pregnancy, in the previous year and the billing period with the index consultation for LBP.

### Propensity-score and matching

Differences in observed patient level characteristics between intervention group (with SMT) and control group (without SMT) could bias estimates of the treatment effects. We used a propensity score matching, stratified by gender, to adjust for treatment assignment. A logistic regression model was used to determine the propensity score, which was in our study the assignment probability to SMT based on 18 predictors as described below. We performed a 1:1 nearest neighbor matching on the logit of the propensity score using calipers of width equal to 0.2 of the standard deviation of the logit of the propensity score. Choice of caliper width followed research done by Austin, who showed that this caliper width adequately reduces the mean square error of the resulting estimation of treatment effect, when some of the dependent covariates are continuous [21].

We calculated four separate propensity-score matchings for the following groups: (1) all patients with SMT and acute LBP, (2) subgroup of insured members entitled to sick leave, (3) patients who only consulted a GP, and (4) patients who only consulted orthopedic surgeon (OS).

### Matching criteria

For matching we used the following variables of relevance for treatment assignment and the outcomes of interest based on the patient history in the 12 months period before the manual therapy. As demographic variables we used age, gender, living in eastern or western Germany and insurance status (insured as dependent, insured as member, or as retiree). The following regional context variables were used: population density, median earnings, rate of employees without graduation, and unemployment rate. To match for healthcare utilization we used the type of doctor that performed manual therapy (family doctor, orthopedic specialist or doctor from other specialty), number of hospital admissions, and number of different prescribed pharmaceutical substances. For balancing comorbidity we used the Charlson Comorbidity Index [22] and additionally the following comorbidities as dummy coded variables (yes/no): anxiety, depression, migraine, addiction to alcohol, somatoform disorder, and bipolar disorder.

### Check for balance

We used the standardized difference in mean (SDM) to compare the distribution of baseline covariates between treatment group and control group.

### Outcome measures

Our primary outcome was documentation of a new consultation for LBP within the next four three-monthly billing periods (one year) as a surrogate parameter for continuous or recurrent LBP and days of sick leave for LBP. Number of visits to a specific provider cannot be determined with health claims data due to capitation. Sick leave for other reasons was disregarded but is reported. Sick leave can only be analyzed for insured members since dependent members (housewives, dependent children up to 25 years) and retirees can officially not be on sick leave. Since we are restricted to three month billing period the number of days within the index period could vary among patients in both groups.

Secondary outcomes were utilization of health services for LBP like physiotherapy, pain medication, acupuncture, imaging, and total health care costs in the billing period following the index period the next 12 month (4 billing periods). Costs are reported from the perspective of the health insurance. We report total health care costs, which is in our analysis the sum of: costs for hospital admissions, outpatient care, medication costs, costs for care provided by nonmedical practitioners (e.g. physiotherapists and occupational therapists), and medical aids.

### Statistics

We performed a univariate analysis to compare the outcome parameters between the matched intervention group and control group. A pre-index period of 4 calendar quarters (previous year) was used to establish balance between the groups. Outcomes were reported for (1) the index quarter which was defined by the first calendar quarter with a LBP consultation in 2011 (index period), (2) the period of one quarter after the index period (following period) to analyze short term effects, and (3) the period of 4 calendar quarters (following year) after the index period.

For count variables we reported the mean and confidence intervals (CI), unless otherwise specified. The categorical data are reported as percentages. Due to the large sample size, statistical tests demonstrated almost always significant difference between the groups. To better judge the differences we report standardized effect sizes for all outcome measures (SMD). The data were analyzed using R [23] and the “MatchIt” package [24] was used for matching [25].

## Results

### Population and patient selection

We identified a total of 113,652 adult patients with acute LBP of whom 21,020 (18%) received SMT by physicians out of a source population of 3.859.239 people who consulted at least once a physician for any reason in 2011 **(Fig 1)**. After excluding patients fulfilling exclusion criteria, like pain in the cervical or thoracic spine (n = 412,495) or chronic back pain (n = 111,470), we were able to match 17.965 patients. A total of 3055 patients who received SMT could not be matched due to incomplete data. A total of 7.179 patients were only treated by orthopedic surgeons and 7.266 only by GPs in the index billing period.

### Patient characteristics and balance

**Fig 2:**
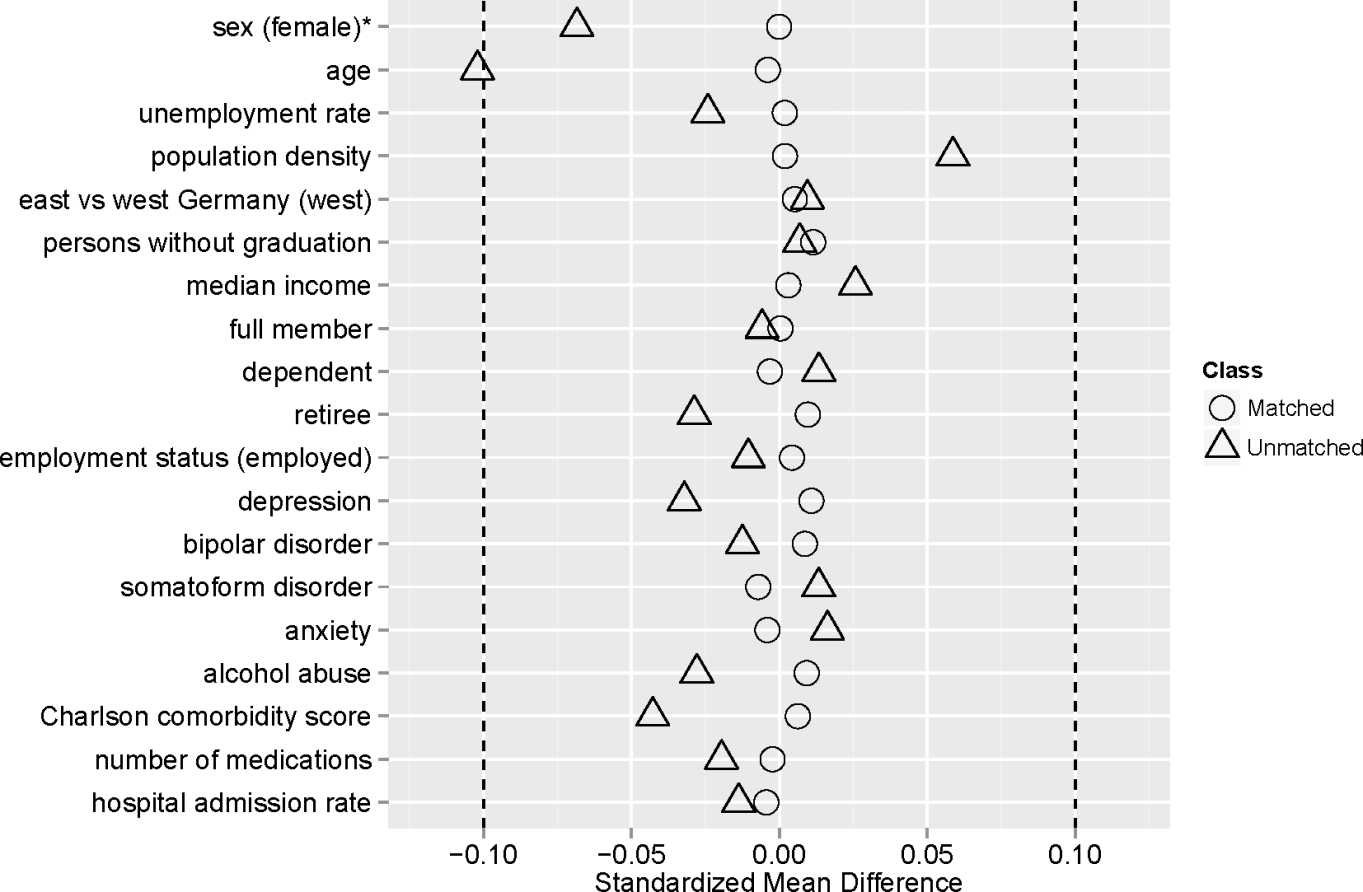
Absolute standardized differences before and after propensity score matching comparing covariate values for patients receiving spinal manipulation therapy and controls.

The average age of patients treated with SMT and controls was 36.3 years and 49.5% were female. Characteristics and comorbidities after matching are shown in **Table 1**. A good balance was achieved **(Fig 2)**.

**Table 1:** Description of the matching variables of the intervention and control group.

	patients with SMT n = 17.965	Controls n = 17.965	SMD (95% CI)
sex (% female)*	49.5%	49.5%	0
age (mean)	36.3	36.3	-0.004 (-0.024; 0.017)
**regional data based on patients residence**			
unemployment rate	7.3%	7.2%	0.002 (-0.019;0.023)
population density / km^2^	942.2 (926.7;957.7)	940.1	0.002 (-0,019;0,023)
east vs. west Germany (% west)	92.6%	92.5%	0.005 (-0.015;0.026)
proportion of persons without graduation from school	14.8%	14.7%	0.011 (-0.009;0.032)
median income (in €)	2789.7	2788.6	0.003 (-0.018;0.024)
**individual health claim data**			
insurance status			
■ full member (%)	85.7%	85.7%	(-0.020;0.021)
■ dependent (%)	13.4%	13.5%	-0.003 (-0.024;0.018)
■ retired (%)	0.8%	0.8%	0.010 (-0.011;0.030)
employment status (% employed)	80.3%	80.1%	0.004 (-0.016;0.025)
depression (%)	11.7%	11.3%	0.011 (-0.010;0.032)
bipolar disorder (%)	0.3%	0.2%	0.009 (-0.012;0.029)
anxiety (%)	6.9%	7.0%	-0.004 (-0.025;0.017)
migraine (%)	6.4%	6.5%	-0.006 (-0.027;0.015)
somatoform disorder (%)	12.0%	12.2%	-0.007 (-0.028;0.014)
alcohol abuse (%)	1.2%	1.1%	0.009 (-0.011;0.030)
Charlson comorbidity index (mean Score)	0.065	0.063	0.006 (-0.014;0.027)
number of medications in the previous year	2.1	2.1	-0.002 (-0.023;0.018)
hospital admission rate in the previous year	0.098	0.1	-0.004 (-0.025;0.016)
healthcare costs (in €)	926.3	954.9	-0,014 (-0.035;0.007)

* exact matching

SMT = spinal manual therapy

SMD = standardized mean difference

### Effects of SMT on follow up visits and sick leave

There was negligible lower sick leave, SMD -0.003 [95% CI -0.025–0.019]) due to LBP in the index billing and overall sick leave for any cause was lower SMD: -0.017 [95% CI -0.039–0.005]) in the SMT vs. the control group. In the following year (4 billing periods) sick leave for LBP was also slightly lower, SMD: -0.017 [95% CI -0.039–0.005]) (**Table 2**). More than half of the patients (56.4% vs. 56.6%) in both cohorts did not reconsult for LBP within the next year. There was only a minimal difference comparing those who were only seen by orthopedic surgeons (56.9% vs. 56.7% SMD 0.005 [95% CI -0.027–0.038]) compared to those seen only by GPs (57.6% vs. 56.2%, SMD 0.028 [95% CI (-0.005–0.061)]) **(Table 3)**.

**Table 2:** Comparison of outcomes of patients treated with and without spinal manual therapy.

	time period	patients with SMT (95% CI)	Controls (95% CI)	SMD (95% CI)
**sick leave and reconsultation**		n = 15.236*	n = 15.236*	
sick leave for low back pain in days*	previous year	0.01 (0.08;0.12)	0.01 (0.08; 0.12)	-0.038 (-0.060;-0.016)
	**index period**	**1.25 (1.13;1.37)**	**1.27 (1.17;1.37)**	**-0.003 (-0.025; 0.019)**
	following period	0.33 (0.23;0.43)	0.26 (0.20;0.32)	0.010 (-0.012; 0.032)
	following year	0.86 (0.73;0.99)	0.93 (0.81;1.05)	-0.008 (-0.030; 0.014)
sick leave total (any cause) in days*	previous year	7.1 (6.70;7.47)	7.4 (7.08;7.88)	-0.011 (-0.033; 0.011)
	**index period**	**3.6 (3.33;3.80)**	**3.8 (3.49;3.93)**	**-0.017 (-0.039; 0.005)**
	following period	2.4 (2.16;2.58)	2.6 (2.41;2.84)	-0,014 (-0.036; 0.008)
	following year	8.9 (8.49;9.23)	9.4 (9.08;9.85)	-0,025 (-0.047;-0.003)
no reconsultation for low back pain	following year	56.4%	56.6%	-0.005 (-0.026;0.015)
**health service utilization**		n = 17.965	n = 17.965	
imaging for low back pain (total)	**index period**	**30.6%**	**23.0%**	**0.164 (0.143;0.185)**
	following period	4.8%	4.3%	0,026 (0.005;0.046)
	following year	22.9%	19.3%	0.085 (0.064;0.106)
CT or MRT imaging for low back pain	**index period**	**2.5%**	**2.5%**	**0.003 (-0.017;0.024)**
	following period	2.6%	2.1%	0.034 (0.013;0.054)
	following year	8.2%	6.9%	0.048 (0.027;0.069)
physiotherapy	**index period**	**18.5%**	**14.7%**	**0.098 (0.077;0.119)**
	following period	5.6%	4.6%	0.044 (0.023;0.065)
	following year	19.7%	16,2%	0.090 (0.069;0.111)
pain medication in % and DDD	**index period**	**35.2%**	**33.5%**	**0.036 (0.016;0.057)**
		**6.7 DDD**	**6.5 DDD**	**0.018 (-0.003;0.038)**
	following period	7.7%	8.2%	-0.018 (-0.038;0.003)
		1.7 DDD	2.0 DDD	-0.031 (-0.052;-0.010)
	following year	36.0%	36.6%	-0.012 (-0.033;0.009)
		10.3 DDD	11.2 DDD	-0.037 (-0.058;-0.016)
acupuncture	**index period**	**3.6%**	**2.9%**	**0.037 (0.016;0.057)**
	following period	2.0%	1.5%	0.038 (0.018;0.059)
	following year	4.8%	3.8%	0.046 (0.025;0.067)
hospitalizations per person (any cause) and duration in days	**index period**	**0.02**	**0.03**	**-0.049 (-0.069;-0.028)**
		**0.1 (0.08;0.11)**	**0.1 (0.09;0.11)**	**-0.022 (-0.043;-0.001)**
	following period	0.02	0.03	-0.021 (-0.041;0.000)
		0.1 (0.11;0.16)	0.1 (0.12–0.18)	-0.006 (-0.027;0.014)
	following year	0.1	0.1	-0.035 (-0.056;-0.014)
		0.5 (0.44;0.54)	0.5 (0.48;0.56)	-0.008 (-0.029;0.013)

* insured members entitled to sick leave.

SMT = spinal manual therapy

SMD = standardized mean difference

DDD = daily defined doses

CI = confidence interval

**Table 3:** Comparison between patients only seen by general practitioners (GP) or only orthopedic surgeons in the index period (OS).

	time period	patients with SMT		controls		SMD	
						SMT patients vs. controls	
		GP only	OS only	GP only	OS only	GP only (CI 95%))	OS only (CI 95%))
n		n = 7.179*; n = 6.253**	n = 7.226*; n = 5.993**	n = 7.179*; n = 6.253**	n = 7.226*; n = 5.993**		
no reconsultation for low back pain(%)*	following year	57.6%	56.9%	56.2%	56.7%	0.028 (-0.005; 0.061)	0.005 (-0.027; 0.038)
sick leave for LBP days (d) (CI 95%)**	**index period**	**1.4 d (1.23–1.58)**	**0.7 d (0.58–0.77)**	**1.5 d (1.32–1.56)**	**0.7 d (0.57–0.77)**	**-0.008 (-0.043; 0.028)**	**0.002 (-0.034; 0.038)**
	following year	0.8 d (0.65–1.03)	0.8 d (0.62–0.99)	1.2 d (1.07–1.46)	0.7 d (0.53–0.84)	-0.048 (-0.084;-0.014)	0.017 (-0.019; 0.053)
total cost (without sick benefit pay) (€)*	index period	246.9 €	315.4 €	225.3 €	335.8 €	0.042 (0.009; 0.074)	-0.027 (-0.060; 0.006)
	following year	1083.4 €	1198.0 €	970.3 €	1285.4 €	0.033 (0.000; 0.066)	-0.027 (-0.060; 0.006)
imaging for (%) low back pain (total)	**index period**	**15.1%**	**41.3%**	**5.6%**	**36.7%**	**0.265 (0.233; 0.298)**	**0.093 (0.060; 0.125)**
	following year	17.2%	26.6%	13.7%	24.0%	0.093 (0.060; 0.125)	0.059 (0.026; 0.092)
CT or MRT imaging for low back pain	**index period**	**1.7%**	**2.9%**	**1.2%**	**3.3%**	**0.039 (0.006;0.071)**	**-0.018 (-0.051;0.014)**
	following year	6.8%	8.6%	5.0%	8.4%	0.076 (0.043;0.109)	0.006 (-0.027;0.039)
physiotherapy for low back pain (%)*	**index period**	**15.1%**	**20.2%**	**10.9%**	**17.2%**	**0.118 (0.085; 0.151)**	**0.075 (0.042; 0.108)**
	following year	17.8%	20.2%	13.8%	17.8%	0.105 (0.072; 0.137)	0.061 (0.029; 0.094)
pain medication (%)*	**index period**	**37.3%**	**31.1%**	**36.9%**	**27.7%**	**0.008 (0.025; 0.041)**	**0.074 (0.042; 0.107)**
	following year	36.7%	34.6%	37.0%	34.4%	-0.005 (-0.038; 0.028)	0.005 (-0.027; 0.038)
acupuncture (%)*	**index period**	**2.5%**	**4.4%**	**1.7%**	**4.2%**	**0.056 (0.023; 0.089)**	**0.010 (-0.023; 0.042)**
	following year	3.5%	5.6%	2.5%	5.7%	0.053 (0.020; 0.085)	-0.005 (-0.038; 0.027)
referral to orthopedic surgery (%)*	following period	7.2%	n.a.	5.6%	n.a.	0.063 (0.031; 0.096)	n.a.
	following year	22.4%	n.a.	20.2%	n.a.	0.054 (0.021; 0.087)	

Values for sick leave, imaging, physiotherapy, pain medication acupuncture for LBP, are zero in all groups due to the selection of patients with new diagnosed LBP and no LBP diagnose in the 2 quarters before the index period.

* all patients

** patients entitled to sick leave

LBP low back pain

SMT = spinal manual therapy

SMD = standardized mean difference

n.a. = not applicable

CI = confidence interval

### Effects of SMT on health service utilization

SMT was associated with a higher proportion of imaging of (30.6% vs. 23%, SMD: 0.164 [95% CI 0.143–0.185]) in the index billing period (Table 2), mainly plain x-ray. The effect was relatively more pronounced in patients only seen by GPs (15.1% vs. 5.6%, SMD 0.265 [95% CI 0.233–0.298]) **(Table 3)** compared to orthopedic surgeons (41.3% vs. 36.7%, SMD 0.093 [95% CI 0.060–0.125]). In the following year there was only a small difference (< 5%points) in imaging procedures for LBP between SMT patients and controls. Mean differences between SMT patients and controls were under 5% points for pain medication, acupuncture, and physiotherapy prescriptions **(Table 2)**. Patients seen exclusively by orthopedic surgeons received more physiotherapy and more acupuncture at baseline and follow up in both groups compared to GPs. Overall SMT patients received more medical services (Table 2). However differences between SMT patients and controls were generally larger in patients seen by GPs. GPs provision of SMT did not affect the number of referrals to orthopedic surgery in the following year **(Table 3)**.

### Effects of SMT on health care costs

The total health care costs (without sick pay benefits) in the index billing period were marginally lower in the SMT group (288 € versus 297 €, SMD -0.014 [95% CI -0.035–0.007]), mainly due to lower costs for hospitalization and sick leave, despite higher costs for imaging and physiotherapy **(Table 4)**. In the following year the costs remained lower again mainly due to a lower rate of hospitalization. Due to capitation in the ambulatory setting it is not possible to separate accurately costs for LBP from costs for other disorders.

**Table 4:** Average costs for health services.

	time period	patients with SMT	controls	SMD (CI-95%)
		n = 17.965; n = 15.236*	n = 17.965; n = 15.236*	
total (without sick benefit pay)	**index period**	288.7 €	297.6 €	-0.014 (-0.035;0.007)
	following period	194.1 €	204.2 €	-0.011 (-0.032;0.009)
	following year	1113.8 €	1157.5 €	-0.007 (-0.028;0.013)
sick pay benefits*	**index period**	27.0 €	28.5 €	-0.003 (-0.024;0.018)
	following period	19.0 €	18.0 €	0.005 (-0.027; 0.038)
	following year	78.2 €	89.1 €	-0.016 (-0.038; 0.006)
imaging (total)	**index period**	7.4 €	6.1 €	0.066 (0.045;0.086)
	following period	3.4 €	2.9 €	0.029 (0.008;0.050)
	following year	13.1 €	11.0 €	0.053 (0.032;0.073)
physiotherapy	**index period**	19.2 €	15.0 €	0.094 (0.074;0.115)
	following period	6.2 €	5.2 €	0.036 (0.015;0.056)
	following year	29.2 €	24.0 €	0.067 (0.046;0.088)
pain medication	**index period**	5.4 €	5.0 €	0.036 (0.015;0.056)
	following period	1.3 €	1.5 €	-0.022 (-0.043;-0.002)
	following year	8.4 €	8.7 €	-0.010 (-0.031;0.010)
hospitalization (any cause)	**index period**	41.4 €	57.1 €	-0.041 (-0.062;-0.021)
	following period	58.4 €	62.1 €	-0.006 (-0.026;0.015)
	following year	222.8 €	249.0 €	-0.019 (-0.040;0.002)

* insured members entitled to sick leave

SMT = spinal manual therapy

SMD = standardized mean difference

## Discussion

### Summary of the main results

The provision of SMT for acute non-specific LBP had no relevant impact on follow up visits and days of sick leave for LBP in the index billing period and the following year. SMT was associated with an overall substantially higher proportion of imaging studies for LBP, particularly for patients seen by GPs. SMT did not replace the use of other health services like physiotherapy, acupuncture or prescriptions for pain medication. Overall costs were negligibly lower (≈ 1%) from the health insurance perspective, which was mainly due to lower costs for hospitalization mostly not related to LBP and lower sick pay benefits.

### Meaning of the results

LBP is frequently a recurrent or relapsing condition [20]. It is unlikely that a relatively short intervention of one or two sessions of SMT has a sustained effect on recurrent LBP over a longer period of time. In most clinical trials patients received at least 4 sessions of SMT [7, 9]. Since physicians can only bill twice within a billing period the quantity of provided SMT might be to archive meaningful clinical effects. However, in short term, one might have expected less days of sick leave due to more effective pain relief.

For physicians providing SMT this is a rather disappointing result, which needs to be interpreted carefully. We could only assess outcomes available from health claims data as surrogate parameters for clinical effectiveness. Patient relevant outcomes like pain, functional impairment and treatment satisfaction were not available. We assume that a large proportion of patients receiving SMT did not specifically seek a physician providing SMT. It has been shown that self-selection to a specific therapy for LBP does not lead to better results but increased satisfaction, therefore self-selection should not have a relevant impact on our findings [26]. It is frequently assumed that there is a subgroup of patients who are more likely to benefit from SMT [27] and a clinical prediction rule to select most suitable patients has been developed [15] and validated independently [16, 17, 18]. Our data suggest that physicians in routine care might not successfully select the most suitable patients for SMT. Implementation of existing rules and more research to help clinicians in selecting patients who are most likely to benefit from SMT is needed. It is also likely that quality of provided SMT is subject to large differences between the providers, which might also explain why we were unable to find relevant effect on the available endpoints. By international standards the training in SMT in Germany of 320 h seems rather short compared to the 1.295 h suggested by the WHO for training chiropractors with various educational backgrounds [28]. Comparing the quantity of training the high prerequisites for certification for physicians need to be taken into account Therefore insufficient training, insufficient quantity and lack of control of adequate provision of SMT are possible explanations for the lack of effectiveness of SMT provided by physicians we observed in our study.

We identified a large proportion (30%) of patients with coded formal contraindication for SMT, e.g. lumbar disc herniation (**Fig 1**), which we excluded from the analysis. Although the common assumption that patients with disc prolapse should not be treated with SMT has been challenged, neither safety nor efficacy of SMT for those patients has been established [29]. This study was not designed to assess safety of SMT, but we have no indication that provision of SMT leads to increased harms, e.g. hospital admissions, based on the available health claims data.

### Health service utilization

In the absence of “red flags” warning signs, which are considered to be a contraindication for SMT, imaging is not useful to improve management of patients with LBP [30]. Consequently, LBP guidelines do not recommend imaging for acute LBP [3, *4, 5, 6*]. We observed a considerably higher rate of inappropriate imaging for patients receiving SMT in our sample with no coded red flags. Traditionally, imaging prior to SMT was advocated by manual therapists. The German medical societies for Manual Therapy have been involved in the development of the national LBP guideline [6]. However, unlike in France [11], there is no official statement regarding imaging prior to SMT. Since imaging has potentially negative effects on the outcome of LBP [31] and is unnecessarily increasing health care costs and exposure to radiation this should be addressed.

We hypothesized that SMT would decrease prescription for physiotherapy. In fact, we observed a small increase. Exercise therapy is considered not effective for acute LBP but for chronic back problems [32].

We hypothesized that SMT would lead to a decreased utilization of acupuncture assuming providers and patients would limit themselves to one form of complementary alternative medicine. Acupuncture is very popular in Germany and covered by statutory health insurance for chronic LBP since 2006 [33]. Only relatively few patients received acupuncture since our patient selection excluded patients with chronic LBP. Roughly half of ambulatory orthopedic surgeons and 8% of GPs are certified and can bill such services, many provide both services. Combination of both services has neither been advocated nor refuted. Despite our assumption there was even a slightly higher utilization of acupuncture.

SMT has the potential for immediate pain relief. We therefore hypothesized that SMT would lead to less filled prescriptions for pain medication. However this was not the case suggesting that pain relief from SMT was not effective or sustained. A limitation is that we have no data on over-the-counter pain medication and pain reported by patients.

### SMT and provided by GPs versus orthopedic surgeon

In absence of a primary care based health system patients have unrestricted access to ambulatory specialist care in Germany. Most (90%) patients with LBP are still seen by GPs, where patients tend to be older and have more comorbidities [33]. Patients treated only by orthopedic surgeons did not have a lower rate of follow up visits. The increase in imaging in patients with SMT was more pronounced in patients seen by GPs. However the overall proportion of imaging by orthopedic surgeons was extremely high in both groups in this low risk group with no coded red flags. Unlike GPs who need to refer patients for imaging, orthopedic surgeons run their own imaging facilities and they might have stronger conviction about biomechanical factors in the etiology of LBP [34]. The observed differences in prescription of physiotherapy reflect the differences in budget for those prescriptions between GPs and orthopedic surgeons.

### Costs

Total costs of patients receiving SMT were only marginally lower in the index billing period (≈ 9 €, ≈ 1%) and in the following year (≈ 40 €, ≈ 1%) from the health insurance providers’ perspective (**Table 4**). This is surprising given the higher rate of imaging and no replacement of any other health services for LBP in the SMT group. Looking in more detail at the lower costs in the SMT group, they are mainly due to lower costs for hospitalization which are mostly not related to LBP. It seems that physicians providing SMT selected healthier subjects for SMT based on information not available in the health claims data. The slightly lower total costs and average sick leave in the billing period prior to the index billing period of patients receiving SMT support this assumption (**Tables 2 and 4**). Our analysis cannot be compared directly to the review of cost effectiveness of SMT which found SMT to be cost-effective [12]. This review included data from patients managed within clinical trials while we report on health claims data from patients in routine care and cost for hospitalization and sick pay could not be directly linked to LBP.

### Strengths and limitations

This is to our knowledge the largest cohort control study on effect of SMT on sick leave, health service utilization and healthcare cost in routine care based on health claims data in younger adults. Using health claims data allows assessing treatment effects on surrogate parameters for clinical effectiveness in routine care. However the available health claim data does not include data on patient relevant outcomes like pain, disability or treatment satisfaction. Despite the fact that we achieved good balance of covariates available in health claim data between the treatment and control group (**Fig 2**), we cannot be certain, that patients in both groups have the same characteristics, for example regarding yellow flags or the pain characteristics such as duration, intensity or number of previous episodes not available in our data base. Therefore, residual confounding cannot be excluded. Manual therapy is a complex not well defined intervention. We could not differentiate manipulations and mobilizations. The available health claims data does not allow any inferences on the quality and the intensity of the provided SMT services. We cannot exclude that patients received SMT either from physicians not certified to bill for SMT or from chiropractors, from private providers or billing fraud, which might have reduced treatment effects. We could not differentiate if physiotherapy prescriptions were made for exercise therapy or manual therapy. Due to miscoding we might have excluded unnecessarily many patients from the analysis, but it is unlikely that miscoding was more frequent in one of the groups.

## Conclusions

SMT for acute LBP provided by physicians in routine care did not clinically meaningful reduce sick leave and reconsultation rates compared to no SMT. SMT did not lead to savings by replacing other health services from the health insurance perspective. This does not prove that SMT is ineffective but might reflect a problem with selection of suitable patients and the quality and quantity of provision of SMT in routine care in Germany. SMT increased imaging studies significantly in population with no coded red flags therefore national Manual Medicine societies should point out more clearly that imaging is not routinely needed prior to SMT. Monitoring of the quality of provided SMT services should be considered.

## Abbreviations

LBP: low back pain
SMD: Standardized mean difference
SMT: spinal manual therapy
